# Beyond hemostasis: a narrative review on the multifaceted therapeutic potential of Ankaferd Blood Stopper

**DOI:** 10.1097/MS9.0000000000004388

**Published:** 2025-11-26

**Authors:** Salima E. Tibi, Muhammad Hashir Nazir, Saleha Nazir, Bhumi Daishik Patel, Gayatri Misra, Tirath Patel, Fatima Nasir, Muhammad Farhan, Nana Sardarova, Nikhilesh Anand

**Affiliations:** a Department of Medicine, Sheba Medical Center, Taybeh Hamerkaz, Israel; b Department of Medicine, King Edward Medical University, Lahore, Pakistan; c Department of Medicine, Windsor University School of Medicine, Cayon, Saint Kitts and Nevis; d Department of Medicine, American University of Antigua, St. John, Antigua and Barbuda; e Department of Medicine, Trinity Medical Sciences University School of Medicine, Kingstown, Saint Vincent and the Grenadines; f Department of Medicine, Akhtar Saeed Medical and Dental College, Lahore, Pakistan; g College of Medicine, Ajman University, Ajman, United Arab Emirates; h Internal Medicine Resident, Henry Ford Warrer Hospital, Warren, Michigan, USA; i Department of Medical Education, University of Texas Rio Grande Valley, Edinburg, Texas, USA

**Keywords:** Ankaferd Blood Stopper, bleeding control, clinical efficacy, hemostatic agent, platelet aggregation

## Abstract

**Background:**

Effective hemorrhage control is essential in managing trauma and surgical patients, with uncontrolled bleeding being a leading cause of mortality. Ankaferd Blood Stopper (ABS), a hemostatic agent historically used in Anatolia, has gained recognition for its ability to modulate red blood cell–fibrinogen interactions to form a stable protein network for rapid bleeding cessation.

**Objective:**

This review explores the multifaceted applications of ABS, evaluating its efficacy, safety, and potential therapeutic roles across various medical fields, including hemostasis, wound healing, antimicrobial activity, and antineoplastic effects.

**Methods:**

A comprehensive synthesis of clinical trials, case reports, and experimental studies was conducted to assess the performance and implications of ABS. This narrative review highlights its application in trauma, surgery, dental procedures, and other clinical contexts, and compares its efficacy with that of established hemostatic agents.

**Results:**

ABS demonstrates significant efficacy in achieving hemostasis in diverse clinical settings, particularly in patients with coagulopathies. Its wound healing and antimicrobial properties enhance its therapeutic versatility. Neurotoxic effects of ABS are also reported, with recent studies providing mixed evidence on nerve safety in animal models. Emerging evidence suggests potential antineoplastic effects, with studies reporting apoptosis induction in cancer cells and protective effects in experimental models.

**Conclusion:**

ABS is a promising hemostatic agent with applications in bleeding control, wound healing, and infection management. While it has shown efficacy in various clinical settings, its safety profile remains a subject of debate, with some studies confirming its biocompatibility and others reporting potential neurotoxic effects. Further large-scale human studies are needed to clarify its long-term safety and establish standardized clinical guidelines for its use.

## Introduction

Uncontrolled bleeding remains a leading cause of mortality in trauma and surgical patients, particularly in prehospital and emergency settings, where swift intervention is critical. The systemic effects of trauma-induced hemorrhage, including coagulopathy and multiorgan failure, highlight the need for effective and accessible hemostatic agents^[1]^. Although various local hemostatic products such as fibrin glues, chitosan-based polymers (Celox), and microporous hydrogel-based sealants (BioHemostat) have been developed, the ideal hemostatic agent must be effective, safe, cost-effective, and easy to use^[1,2]^.

**HIGHLIGHTS**

- ABS rapidly achieves hemostasis via a unique red blood cell–fibrinogen interaction.
- Demonstrates wound healing, antimicrobial, and emerging antineoplastic properties.
- Effective in controlling bleeding across trauma, surgery, and dental procedures.
- Comparable efficacy to other hemostatic agents like chitosan-based products.
- Toxicological assessments affirm ABS’s safety with no significant adverse effects.

The Ankaferd Blood Stopper (ABS) is a hemostatic agent traditionally used in Anatolia for centuries and has been approved by the Turkish Ministry of Health for local applications in dental and superficial bleeding^[3,4]^. Unlike conventional agents, ABS functions by modulating red blood cell–fibrinogen interactions to form a unique protein network that halts bleeding^[4]^. Its clinical applications span a broad spectrum, including gastrointestinal (GI), dental, and surgical bleeding, as evidenced by the growing body of case reports and experimental studies.

Interest in ABS has expanded significantly in recent years, driven by a surge in clinical and experimental research highlighting its versatility. For instance, a 2024 study demonstrated ABS’s effectiveness in managing GI bleeding, achieving acute hemostasis in all treated patients, suggesting potential superiority in less experienced hands^[5]^. Additionally, a July 2025 technical review by the European Society of Gastrointestinal Endoscopy reported that ABS achieved 73–100% immediate hemostasis across diverse GI bleeding sources, including peptic ulcers and malignancies^[6]^. Emerging applications beyond hemostasis are also gaining traction; a May 2024 study on ABS-doped nanofiber dressings showed accelerated wound healing in rats without adverse effects^[3]^. Furthermore, recent investigations into its antineoplastic properties, such as a 2023 review on pharmacobiology in neoplastic disorders, indicate ABS’s ability to induce apoptosis in chronic lymphocytic leukemia (CLL) cells at low doses^[7]^. These developments, including multiple clinical reports from 2024 to 2025 and experimental studies, underscore the timeliness of a new synthesis of ABS research, as they reveal evolving insights into its pleiotropic effects and potential in resource-limited or coagulopathy-prone settings.

Despite the increasing use of ABS, there remains a need to systematically explore its potential in diverse clinical scenarios. This review aims to consolidate existing knowledge of the hemostatic, antimicrobial, wound-healing, and antineoplastic properties of ABS. By integrating findings from clinical trials, case reports, and experimental studies, this review aimed to evaluate the safety, efficacy, and broader implications of ABS in clinical practice. This comprehensive understanding could inform its rational integration into modern healthcare and address critical gaps in hemorrhage control and therapeutic interventions. This manuscript is made compliant with the TITAN checklist to ensure transparency in the reporting of Artificial Intelligence^[8]^.

## Methodology

Our team conducted a thorough search of the literature on PubMed, Embase, and Google Scholar for English-language publications on ABS up to October 2025. Keywords included “Ankaferd Blood Stopper,” “Ankaferd Hemostat,” “hemostatic agent,” “wound healing,” “antimicrobial,” “antineoplastic,” and combinations thereof. We screened titles and abstracts to identify relevant original studies (clinical trials, case series, animal experiments, *in vitro* studies) and reviews. Inclusion criteria were peer-reviewed articles addressing therapeutic uses of ABS; we excluded non-English articles, abstracts without complete data, and studies unrelated to ABS’s medical application. Data were extracted qualitatively and organized by application (hemostasis, wound healing, antimicrobial, etc.). This approach is consistent with recent reviews (e.g., Malkan 2022). Data were extracted qualitatively and organized by application (hemostasis, wound healing, antimicrobial, etc.) as described below. To enhance comprehensiveness, we incorporated advanced search operators for date ranges (e.g., 2024–2025) and specific domains (e.g., site:pubmed.ncbi.nlm.nih.gov) to capture the most recent surge in the literature, while ensuring bias control by balancing the inclusion of both positive and negative findings.

## Composition of Ankaferd Blood Stopper

ABS is a standardized mixture of five medicinal plant extracts that have been historically utilized in Anatolia for their hemostatic properties. These extracts include *Thymus vulgaris* (thyme), *Glycyrrhiza glabra* (licorice), *Vitis vinifera* (grapevine), *Alpinia officinarum* (lesser galanga), and *Urtica dioica* (common nettle)^[2,9,10]^. Each plant has unique properties that enhance the effectiveness of ABS as a topical hemostatic agent for managing visible bleeding.

## Components of Ankaferd Blood Stopper

**Figure 1.**
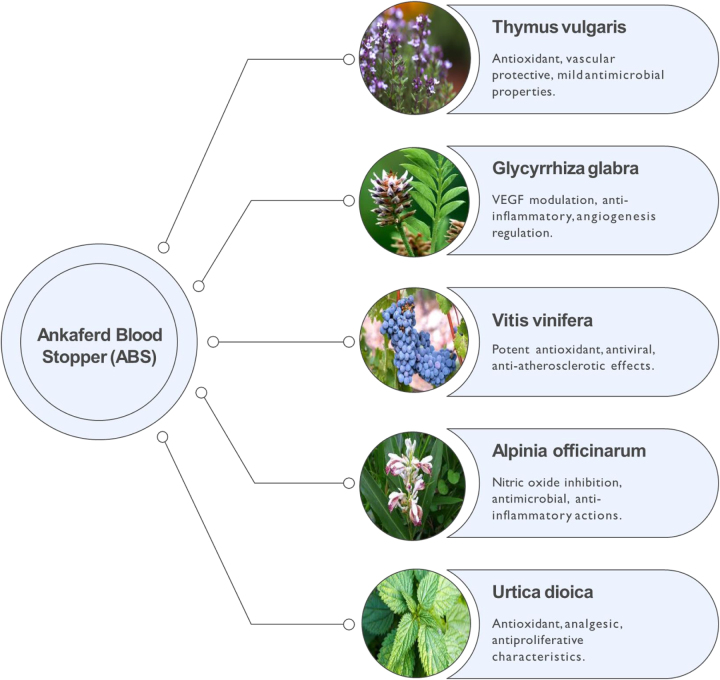
The five herbal components of ABS, *T. vulgaris, G. glabra, V. vinifera, A. officinarum*, and *U. dioica*, each contribute unique antioxidative, anti-inflammatory, antimicrobial, or vascular-stabilizing effects, producing synergistic hemostatic and reparative activity. ABS, Ankaferd Blood Stopper.

The ABS formulation consists of the following quantities of plant extracts per 100 ml of solution^[11]^: *T. vulgaris*: 5 mg, *G. glabra*: 9 mg, *V. vinifera*: 8 mg, *A. officinarum*: 7 mg, and *U. dioica*: 6 mg. This composition allows ABS to act as a potent hemostatic agent by leveraging the combined effects of these plant extracts. Figure 1 illustrates the composition and synergistic actions of the five herbal components.

## Hematological and vascular actions of plant components

*T. vulgaris*: Owing to its antioxidative properties, *T. vulgaris* prevents lipid peroxidation, thereby protecting vascular integrity and reducing oxidative stress^[12]^.

*G. glabra* modulates vascular endothelial growth factor production and inhibits cytokine-induced neovascularization, contributing to vascular stability and reducing hemorrhage risk^[11]^.

*V. vinifera* exhibits potent antioxidant and antiatherosclerotic properties. It also exhibits antiviral effects, further enhancing its utility in wound healing and hemostasis^[13–15]^.

*A. officinarum*. This extract inhibits nitric oxide production, which can mitigate inflammation, support vascular constriction, and promote hemostasis. It also has antimicrobial and antioxidant properties^[16,17]^.

*U. dioica* contains compounds, such as polyphenols and carotenoids, which act as antioxidants. *U. dioica* also has analgesic, antiproliferative, and hypotensive properties^[18]^. ABS derives its hemostatic efficacy from a carefully balanced blend of plant extracts, each contributing distinct pharmacological properties that collectively enhance its ability to efficiently manage bleeding.

## Ankaferd: mechanism of action

**Figure 2.**
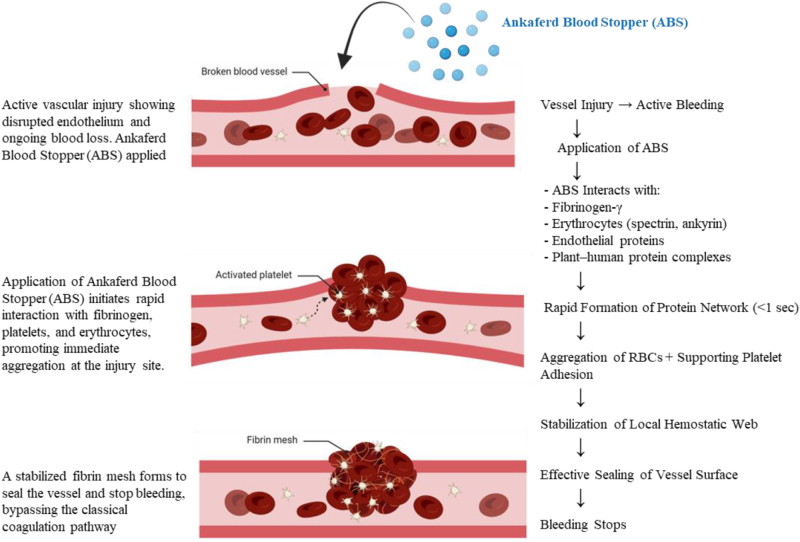
Mechanistic overview of ABS action showing rapid formation of a protein network through fibrinogen gamma and erythrocyte aggregation independent of the classical coagulation cascade. Plant- and human-derived proteins interact synergistically to stabilize clot formation and vascular integrity. ABS, Ankaferd Blood Stopper.

ABS exhibits hemostatic efficacy through the rapid promotion of a complex protein network, specifically involving fibrinogen gamma and erythrocyte aggregation^[4]^. This protein network is a cornerstone of the ABS mechanism, as it supports both primary and secondary hemostasis without directly affecting individual coagulation factors^[19–21]^. Instead, ABS facilitates hemostasis through a distinct pathway involving protein agglutination, erythrocyte aggregation, and interaction with the vascular endothelium. The primary mechanism of ABS is the instantaneous (<1 s) formation of a protein network upon contact with blood^[2]^. This network integrates red blood cells, activated leukocytes, and plasma proteins, thereby providing a scaffold for erythrocyte aggregation. High-resolution scanning electron microscopy demonstrated that ABS forms a stable protein matrix that encapsulates red blood cells, stabilizing the clot through rapid erythroid aggregation^[2]^. Figure 2 illustrates the mechanism of action of ABS.

## Erythrocyte aggrembrane receptors

In the presence of ABS, erythrocyte aggregation occurs via interactions with membrane proteins such as spectrin and ankyrin. This mechanism is central to its action, as it covers the entire physiological hemostatic process without disrupting traditional coagulation cascades. Erythrocyte aggregation is further supported by ABS proteins, including actin depolymerization factor and NADH dehydrogenase^[4]^.

## Plant and human protein interactions

Proteots contain plant-derived proteins, such as NADP-dependent malic enzymes, ATP synthase subunits, and ribulose bisphosphate carboxylase. These proteins interact synergistically with human peptides, including mucin-16 and ATP synthase, highlighting the unique interplay between plant- and human-derived components in the hemostatic process^[19,21]^.

## Effects on coagulation parameters

Although ABS does not alter the levels of coagulation factors II, V, VII, VIII, IX, X, XI, or XIII, it significantly reduces plasma fibrinogen activity and antigen levels, resulting in prolonged thrombin time. This indicates that ABS facilitates clot formation through mechanisms independent of the traditional coagulation cascade^[20]^.

## Resistance and stability of the protein complex

The protein network formed by ABS is highly resistant. It withstands heat and detergents, although it can be broken down by trypsin or sonication, thereby releasing erythrocyte membrane proteins. This stability underscores its effectiveness in promoting robust hemostasis^[22]^.

## Cellular and molecular effects

ABS activates various transcription factors, including AP2, AR, CREB, E2F, NF-κB, and p53. This suggests that ABS may influence multiple intracellular pathways, thereby contributing to its pleiotropic effects^[22]^. Additionally, ABS has demonstrated antiproliferative effects on cancer cells, including breast cancer and leukemia cells, by altering the expression of proteins involved in cell cycle regulation and apoptosis^[23,24]^. Recent proteomic analyses further support these findings, showing ABS’s impact on aberrantly expressed proteins in estrogen receptor-positive breast cancer subtypes^[23]^.

## Clinical applications of Ankaferd Blood Stopper

### Dental and gastrointestinal bleeding control

ABS has demonstrated significant efficacy in various clinical scenarios, particularly in managing bleeding. In dental applications, ABS has shown promise for controlling bleeding in patients with underlying coagulation disorders. Kazancıoğlu *et al* highlighted its effectiveness in patients with hemophilia A who underwent tooth extraction^[25]^. Similarly, Bykul *et al* reported successful outcomes in controlling dental bleeding in individuals with von Willebrand disease and chronic liver failure, further emphasizing its utility in managing bleeding in complex medical conditions^[26]^.

In addition to dental applications, ABS has been successfully used in cases of massive bleeding. Solak *et al* documented the intravesical use of ABS to control severe hematuria in a hemodialysis patient with disseminated intravascular coagulation^[10]^. Another remarkable case presented by Kurt *et al* described a patient with hemorrhagic shock due to massive hematemesis, hematochezia, and epistaxis. The administration of ABS through the oral, rectal, and nasal routes is instrumental in stabilizing the patient and achieving hemostasis^[27]^.

ABS has been particularly effective in the treatment of GI bleeding. Baş *et al* found that ABS, used alone or in combination with other techniques, successfully achieved hemostasis and reduced rebleeding rates in patients with GI tumors, even when applied by less experienced endoscopists^[5]^. Ozaslan *et al* reported rapid bleeding control in patients with peptic ulcer disease following ABS^[28]^. Furthermore, Hacıoğlu documented a unique case of a patient with Glanzmann thrombasthenia and refractory GI bleeding, where oral ABS provided effective resolution after months of unsuccessful medical therapy^[3]^. A recent 2024 study reinforced these findings, showing ABS achieved acute hemostasis in all patients with various GI bleeds, highlighting its role as an additional strategy for less experienced practitioners^[5]^.

### Postoperative bleeding control

ABS has also been explored in postoperative bleeding management. A study on pediatric adenoidectomy cases demonstrated that ABS significantly reduced the duration of bleeding and the need for additional packing compared with saline^[29]^. However, not all the findings were unequivocally positive. A randomized, placebo-controlled study examining ABS use in postoperative breast surgeries noted an increased incidence of infection and wound healing complications in the ABS group, suggesting that its application might carry certain risks under specific circumstances^[30]^. In a double-blind, placebo-controlled, randomized clinical trial involving patients premedicated with clopidogrel and acetylsalicylic acid undergoing emergency coronary artery bypass grafting, the use of ABS significantly reduced postoperative bleeding and the need for blood transfusions. Importantly, no patients in the ABS group required surgical revision due to severe bleeding or cardiac tamponade, highlighting its safety and efficacy in high-risk surgical procedures^[31]^.

These findings underscore the versatility of ABS in hemostasis, making it a valuable adjunct in various clinical settings. However, its application must be tailored to individual patient needs and potential risks.

### Wound healing

ABS has demonstrated potential in promoting wound healing in various clinical and experimental contexts. Topal *et al* conducted a study that highlighted significantly enhanced wound healing and contraction in ABS-treated wounds compared with controls^[32]^. These findings suggest that ABS not only contributes to hemostasis but also aids in healing. In a notable clinical case reported by Octurk *et al*, a female breast cancer patient with an esophageal ulcer showed complete healing within 20 days following endoscopic application of a 10 ml ABS spray^[33]^. A study on nondiabetic and diabetic rat populations found ABS effective in healing gingival wounds in the nondiabetic population^[34]^. A recent May 2024 study compared wound healing by ABS alone with ABS-doped nanofiber wound dressing in the rat population, reporting that ABS-doped nanofiber wound dressing caused rapid wound healing without adverse effects on the process^[35]^. This builds on prior evidence, suggesting ABS’s antioxidant and anti-inflammatory properties may synergize with advanced materials for optimized outcomes^[34]^.

The mechanism underlying the wound-healing effects of ABS is thought to involve its antioxidant and anti-inflammatory properties^[34]^, as evidenced in other studies. These properties, coupled with their hemostatic action, make ABS a promising therapeutic agent for managing challenging wounds and ulcers.

### Antimicrobial activity

The antimicrobial properties of ABS have been extensively evaluated, particularly their efficacy against bacterial pathogens. Fisgin *et al* conducted an *in vitro* study to assess the antimicrobial activity of ABS in 102 clinical isolates of both Gram-positive and Gram-negative bacteria. The study revealed that ABS demonstrated significant activity against all bacteria tested, highlighting its potential as an adjunct in infection control^[36]^. Despite its broad antibacterial efficacy, ABS lacks antifungal activities. A study by Ciftci *et al* found that ABS showed no inhibitory effect against *Candida albicans*, suggesting that its antimicrobial effects are selective for bacterial pathogens^[37]^.

Recent studies have expanded on these findings. A 2022 investigation compared ABS’s antimicrobial activities against hypochlorous acid and chlorhexidine, noting its pleiotropic effects on blood cells and tissue regeneration that aid wound healing^[38]^. Additionally, a 2021 study reaffirmed ABS’s strong antibacterial effects against oral microorganisms, positioning it as a hemostatic with added infection-preventive benefits^[39]^. These findings indicate that ABS could serve as a valuable tool for the management of infections associated with wounds or other clinical settings. However, further studies are needed to explore these mechanisms and evaluate their efficacy *in vivo*.

### Antineoplastic effects

Emerging evidence suggests that ABS may possess significant antineoplastic properties, making them promising candidates for cancer therapy. The apoptotic effects of ABS have been demonstrated in leukemia cells, where it stimulates apoptosis through pathways involving protease-activated receptor 1- and p53-independent p21 regulation. The unique composition of ABS plant extracts is believed to contribute to this apoptotic mechanism^[24]^. Proteomic studies have highlighted the impact of ABS on cancer-related proteins. One study identified its effects on aberrantly expressed proteins in breast cancer, particularly the estrogen receptor-positive (estrogen receptor positive) subtype. Proteins such as chaperones p97, ATP synthase subunit beta, selenium-binding protein 1, protein disulfide isomerase family A member 6, and ribosomal protein S10 pseudogene 5 have been identified as targets, underscoring the molecular basis of their antineoplastic effects^[23]^.

In bladder cancer, an *in vitro* study by Sari *et al* showed that ABS exhibited a significantly greater cytotoxic effect on bladder cancer cells than on controls^[40]^. Similarly, studies on multiple myeloma have demonstrated ABS’s ability to reduce plasmacytoma development and improve survival in animal models, suggesting its potential for both *in vitro* and *in vivo* applications^[41]^.

The systemic effects of ABS have been highlighted in studies on Caco-2 colon cancer cells, where ABS influences cell metabolism and cell cycle pathways. Proteomic results revealed that ABS suppressed various cancer targets and regulatory proteins, further supporting its role as a potential therapeutic agent^[42]^. Recent advancements include a 2023 review on the pharmacobiology of topical ABS in neoplastic disorders, which reported that ABS treatment at doses of 0.5–2 µg/ml inhibited tumor cell proliferation and induced over 50% cell death in B-CLL cells^[7]^. Additionally, an ongoing clinical trial (NCT05438771) is evaluating ABS’s effectiveness in preventing oral mucositis due to chemotherapy in adult cancer patients, potentially bridging preclinical antineoplastic effects to clinical oncology^[43]^. Collectively, these findings emphasize ABS’s multifaceted role in oncology, although clinical trials are warranted to validate its efficacy and safety in human cancer treatment.

Figure 3A summarizes clinical applications of ABS.

**Figure 3A.**
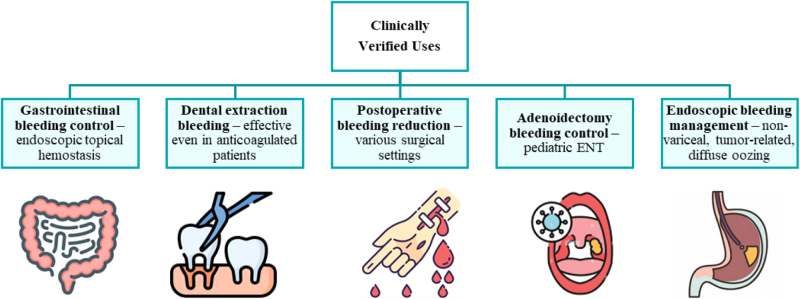
Overview of clinical applications of ABS. ABS, Ankaferd Blood Stopper.

### Findings from animal studies

Animal studies have provided valuable insights into the safety, efficacy, and potential therapeutic applications of ABS. These studies span various domains, including hemostasis, hepatoprotection, nephroprotection, and systemic antioxidant effects.

### Hemostasis in animal models

ABS has demonstrated remarkable hemostatic efficacy in animal models. For instance, a study on rat abdominal aortas successfully achieved hemostasis without any histopathological changes, highlighting its safety^[9]^. Similarly, ABS was used to control bleeding in amputated rat legs treated with warfarin, reducing bleeding by 53.8%. These findings underscore the effectiveness of ABS, even in anticoagulated states^[44]^.

Egemen *et al* examined ABS’s hemostatic effects on the mammalian brain parenchyma and reported significantly higher fibrinogen levels in the ABS-treated group compared with controls. The study concluded that ABS is safe for use in sensitive tissues such as the brain^[45]^.

### Hepatoprotective and nephroprotective effects

ABS also possesses hepatoprotective properties. Studies in rat models of liver ischemia-reperfusion injury demonstrated that ABS reduced oxidative stress and inflammation, thereby protecting the liver^[46]^. ABS exhibited promising results in cases of obstructive jaundice, supporting its role in managing hepatic complications^[47]^.

ABS has shown protective effects against cadmium-induced acute nephrotoxicity in rats. It mitigates oxidative stress and apoptosis mediated by mitochondrial dysfunction, suggesting its potential as a therapeutic agent for kidney injury^[48]^.

### Other applications

The antioxidant and anti-inflammatory properties of ABS extend to various contexts. ABS was found to have a strong protective effect in experimental models of systemic inflammatory conditions, further supporting its versatile therapeutic profile^[47,48]^.

Collectively, animal studies provide a robust foundation for exploring ABS’s clinical applications, particularly in critical and sensitive medical settings. These findings warrant further research on the transition of ABS from experimental to clinical practice.

Figure 3B. summarizes experimental/preclinical applications of ABS.

**Figure 3B.**
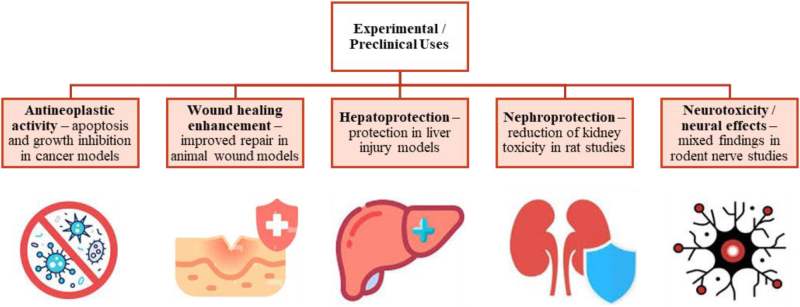
Overview of clinical applications of ABS. ABS, Ankaferd Blood Stopper.

### Comparative efficacy of Ankaferd Blood Stopper and other hemostatic agents

In an experimental rat model, Abacıoğlu found that ABS was as effective as chitosan (Celox) in stopping femoral artery bleeding within 2 min and was significantly more effective than the control group within 4 min^[2]^. Another study by Huri *et al* also found no significant difference in hemostasis time between ABS and Celox, further supporting the comparable effectiveness of these agents^[49]^.

In addition to its hemostatic effects, ABS has shown promise for wound healing. A study assessing the effects of ABS and a tripeptide copper complex (TCC) on wound healing in rats reported that, while the time to observe initial granulation tissue did not differ significantly between treatment groups, both ABS and TCC accelerated the filling of open wounds with granulation tissue and reduced the average healing time^[50]^. A study by Ozmen *et al* compared ABS with ferric sulfate (FS) and formocresol (FC) as pulpotomy agents in primary teeth. It found that there was no significant difference among the groups, suggesting that ABS is as effective as FS and FC^[51]^. An experimental study by Ay *et al* compared the efficacy of ABS with silver sulfadiazine (SSD) in treating partial-thickness burns in rats. It concluded that ABS promotes faster wound healing compared to SSD^[52]^. A recent 2024 comparative evaluation of the erbium, chromium:yttrium scandium gallium garnet laser, FS, and ABS in pulpotomy procedures further confirmed that there were no significant differences in clinical and radiographic success rates, reinforcing ABS’s equivalence in dental applications^[53]^. **Table 1** summarizes the comparative hemostatic efficacy, cost, ease of application, and safety profiles of ABS and other commonly used hemostatic agents.

**Table 1:** Overview of ABS’s hemostatic efficacy, cost, application ease, and safety profile against key alternatives

Agent	Hemostatic efficacy	Cost (approx. USD per unit/treatment)	Ease of application	Safety profile
ABS	High (73–100% immediate hemostasis in GI/surgical bleeds^[5,6]^)	$150–200 (monthly supply/product^[54,55]^)	Topical spray/liquid; rapid (<1 s)	Biocompatible in most studies; potential neurotoxicity in rodents^[62,63]^; no human toxicity reported^[6]^
Chitosan (Celox)	Comparable to ABS (2–4 min femoral bleed stop^[2,49]^)	$10–40 (granules/pouch^[60,61]^)	Granules/gauze; moderate prep	Low allergy risk; biodegradable; minimal adverse events^[2]^
SSD	Good for burns/wounds (faster healing vs. ABS in some^[52]^)	$3–16 (20–50 g tube^[56,57]^)	Cream; easy topical	Antimicrobial; potential skin irritation/allergy^[56]^
FS	Comparable in dental pulpotomy (no sig. diff.^[51,53]^)	$2.50–25 (15–30 ml syringe/solution^[58,59]^)	Solution/gel; simple	Effective; risk of tissue staining/inflammation^[58]^

ABS, Ankaferd Blood Stopper; SSD, silver sulfadiazine; FS, ferric sulfate; GI, gastrointestinal.

Data indicated significant cost disparities. For example, ABS, one clinical report noted a cost of roughly US$150–200 for about 1 month’s supply or a product unit^[54,55]^. By contrast, generic 1% SSD cream, a typical 20–50 g tube, costs only on the order of US$3–16 in the US^[56,57]^. Ferric subsulfate (Monsel’s solution) or FS hemostatic gels are even less costly (around US$2.50–25 per 15–30 ml unit or syringe)^[58,59]^. Chitosan-based dressings (e.g., Celox granules or gauze) are priced at US$10–40 per unit or pouch^[60,61]^. In summary, SSD is by far the lowest-cost agent per treatment, FS is also minimal cost, Celox-level chitosan is moderate, and ABS carries a premium price. The cost comparison is unclear because the price for ABS is given for 1 month’s supply or as bulk, whereas for others, it is listed per unit. The actual cost per treatment can vary significantly across regions and suppliers, but these figures highlight ABS’s higher upfront cost, offset by its multifunctional benefits.

**Figure 4.**
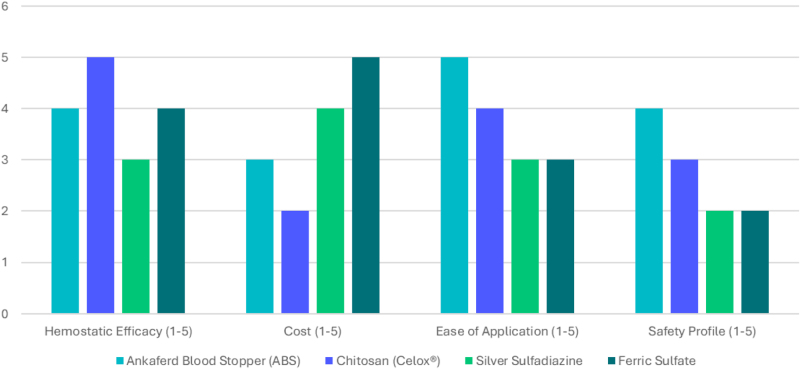
Comparative overview of ABS versus chitosan (Celox), SSD, and FS based on hemostatic efficacy, cost, ease of application, and safety profile, summarizing data from 2016 to 2025 studies. ABS, Ankaferd Blood Stopper; FS, ferric sulfate; SSD, silver sulfadiazine.

Figure 4 presents a bar chart comparing the efficacy, cost, and safety of ABS with those of other hemostatic agents.

### Efficacy and safety profiles

Koluman *et al* demonstrated the toxicological safety of ABS by confirming the absence of harmful substances, such as heavy metals, pesticides, and mycotoxins^[64]^. Further research in a rat model found no evidence of neurotoxicity, with ABS showing no significant impact on neuronal integrity or inflammation compared with controls. These findings support the safety and clinical viability of ABS^[62]^. This contradicts the results of another study that found that the use of ABS in rats caused damage to the myelin sheath and axons of the sciatic nerve^[63]^. To reconcile these discrepancies, a July 2024 study on functional and structural neurodegenerative processes in a mouse sciatic nerve model confirmed ABS’s association with nerve toxicity, including degenerative effects on axons and myelin^[63]^. Additionally, an August 2025 experimental study on rat sciatic nerves evaluated ABS’s effects on nerve conduction, reporting potential impairments that warrant caution in neural applications^[65]^. A 2020 analysis in medulla spinalis surgery, however, found no toxicity, suggesting context-dependent safety^[62]^. Overall, while ABS exhibits biocompatibility in systemic and topical uses and shows no short-term toxicity in oral administration models^[66]^, its neurotoxic potential following direct nerve exposure remains a debated area requiring human studies.

### Drug/agent interactions with Ankaferd Blood Stopper

Available evidence suggests minimal pharmacokinetic interactions of ABS with other drugs. In human hepatocarcinoma (HepG2) cells, ABS did not significantly affect the expression or activity of major cytochrome P450 (CYP) enzymes (CYP1A1, 1A2, 2E1, and 3A4)^[67]^. Thus, ABS is unlikely to alter systemic drug metabolism or cause CYP-mediated drug-drug interactions. A 2023 study on drug interaction potential in hepatocarcinoma cells reinforced this, stating that there is no risk of clinical drug toxicity or metabolic disorders upon human exposure^[68]^.

Pharmacodynamically, ABS maintains efficacy in the presence of anticoagulants. *In vivo* models of anticoagulation demonstrate that ABS shortens bleeding times even when warfarin or heparin is present^[44,69]^. For example, in a rat nasal bleeding model, ABS achieved significantly faster hemostasis than saline, both with and without heparin pretreatment^[69]^. Similarly, ABS controlled bleeding from the femoral artery in warfarinized rats^[44]^. These results imply that ABS’s mechanism (rapid protein agglutination) is effective regardless of systemic coagulation status. In practical terms, no adverse interactions have been reported when ABS is used with other hemostatic agents or anticoagulants. No toxicity or allergic events related to ABS have been reported in human use^[6]^. Overall, we found no evidence of harmful pharmacodynamic or pharmacokinetic interactions between ABS and common drugs in the literature.

### Clinical vs. experimental applications

We have clarified in the text which ABS uses are clinically established versus experimental. Clinically verified uses of ABS include control of surgical and endoscopic bleeding, for example, GI ulcer bleeds, epistaxis, and postsurgical hemorrhage, as documented in human studies^[6,69]^. These applications (often in emergency or intraoperative settings) have demonstrated consistent success, leading to ABS’s approval for hemostasis in Turkey. In contrast, other reported actions of ABS remain preclinical. For instance, ABS’s effects on wound healing and cancer cells have been demonstrated only in animal or *in vitro* models^[70]^. As noted, experiments in rats suggest accelerated burn-healing and antitumor activity^[70]^, but no large-scale clinical trials have yet been conducted. Recent trials, such as NCT05438771, evaluating ABS for chemotherapy-induced mucositis, may help bridge this gap^[43]^.

### Future directions for Ankaferd Blood Stopper

The future of the ABS lies in expanding its applications and understanding its mechanisms through robust clinical and experimental research. While existing studies highlight its efficacy and safety in hemostasis, wound healing, and antimicrobial activity, further exploration of its antineoplastic potential could unlock new therapeutic avenues. Large-scale randomized controlled trials on the human population are essential to validate its long-term effectiveness across diverse clinical scenarios, including its use in coagulopathies, trauma management, and minimally invasive surgery. The data on the neurotoxic effects of ABS is lacking and requires further evaluation in order to explain the safety threshold of ABS, particularly given recent 2024–2025 animal studies showing degenerative nerve impacts^[63,65]^. Additionally, advancements in the molecular profiling of ABS’s bioactive components may lead to the development of targeted formulations optimized for specific medical conditions. Integration into prehospital and emergency care protocols, along with cost-effectiveness analyses, can enhance its accessibility and utility worldwide.

Figure 5A summarizes the timeline of ABS research from 2008 to 2025, highlighting key milestones in clinical, experimental, and translational studies, including ongoing clinical trials exploring new therapeutic indications.

**Figure 5A.**
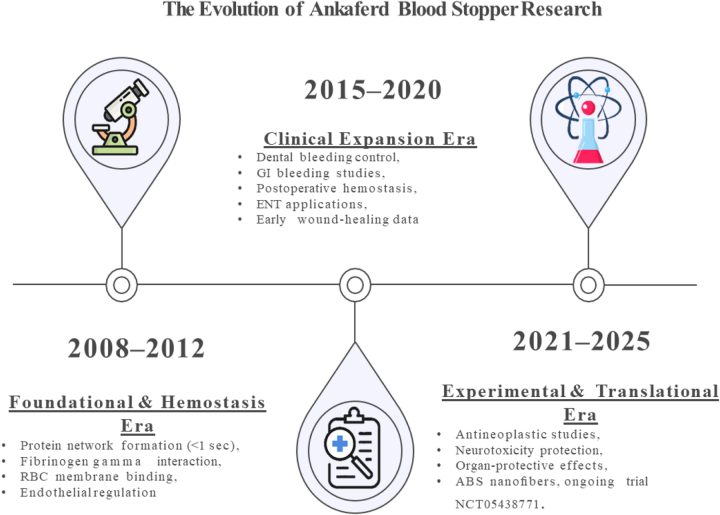
Timeline summarizing the evolution of ABS research from 2008 to 2025. ABS, Ankaferd Blood Stopper.

### Limitations

While ABS is well established as a topical hemostatic agent in clinical practice, evidence for its broader pharmacologic effects remains preliminary. Clinical studies have confirmed ABS’s effectiveness in achieving rapid bleeding control in settings such as traumatic lacerations and during endoscopic procedures^[5,71]^. By contrast, reports of antineoplastic or other therapeutic activities of ABS come almost exclusively from laboratory or animal studies and have not been validated in patients. For example, ABS has been shown *in vitro* to inhibit the growth of various cancer cell lines^[70]^, but no controlled human trials of any anticancer benefit have been performed. Likewise, potential adverse effects, such as neurotoxicity, have only been observed in rodent models^[63,65]^ and have not been studied in humans, leaving the safety profile of ABS in non-hemostatic contexts largely undefined. In short, most proposed uses of ABS beyond bleeding control remain experimental and unproven in clinical trials. We therefore emphasize that claims of multifaceted therapeutic benefits should be viewed as tentative. Well-designed, large-scale clinical studies are needed to rigorously assess ABS’s efficacy and safety in any new indication. At present, ABS’s only indication supported by robust clinical data is hemostasis in surgery and endoscopy^[5,71]^. Until further evidence is available, non-hemostatic applications of ABS must be regarded as investigational.

Figure 5B presents a proposed translational pipeline for future ABS research, highlighting key gaps that require large-scale human studies before clinical adoption of non-hemostatic applications.

**Figure 5B.**
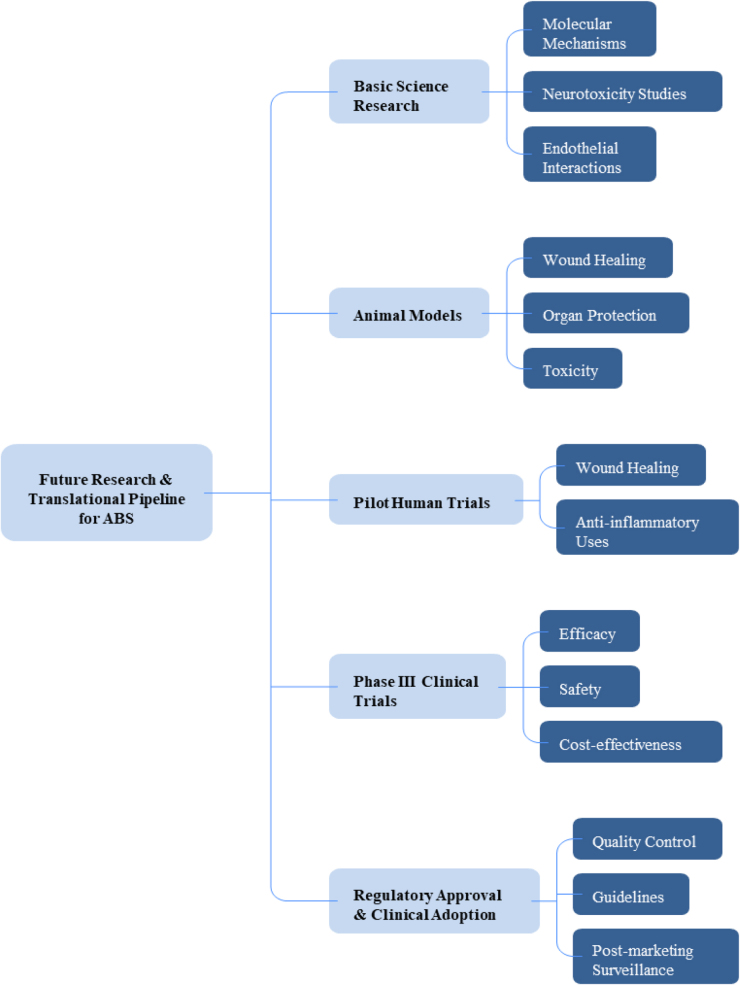
Proposed translational pipeline for future ABS research.

## Conclusion

ABS represents a promising hemostatic agent with established clinical efficacy in bleeding control and emerging potential in wound healing, antimicrobial, and antineoplastic research. Its bioactive plant components and antioxidant mechanisms may contribute to these broader effects, though most evidence remains preclinical. Recent investigations have also underscored the importance of assessing its neurotoxicity profile in animal models. While ABS continues to demonstrate safety and effectiveness in topical hemostatic applications, all proposed non-hemostatic uses should be regarded as experimental until validated by well-designed, large-scale human studies. Continued translational research and controlled clinical trials will be critical to define the true therapeutic scope, safety thresholds, and long-term applicability of ABS in modern medicine.

## Data Availability

All data used in this narrative review are publicly available and sourced from previously published studies. No new data were generated for this work. All included articles have been appropriately cited within the manuscript and are available through the references section.

## Research registration unique identifying number (UIN)

The Research registration unique identification number (UIN) was not required because it is a narrative review.
